# Dental Pathology and Surgery—Salter

**Published:** 1875-02

**Authors:** 


Dental Pathology and Surgery. By S. James A. Salter, M.B., F.R.S., Member of the Royal College of Surgeons, and Examiner in Dental Surgery at the College; Dental Surgeon to Guy's Hospital, London. New York: William Wood & Co., publishers, 27 Great Jones street. 1875. Octavo, cloth, pp. 399. Price $4.50.

The field selected by the author for his work lies upon a common boundary line between surgery and dentistry. It may, with almost equal propriety, be designated either the field of dental surgery or the field of surgical dentistry. It comprises in its scope all of dental surgery which a practical surgeon would desire in the prosecution of his own specialty.

In a rather hasty sketching through the work we are favorably impressed with the chapters upon dental deficiencies and irregularities and the means of remedy, and the various means of remedying deficiencies of the hard and soft palates by the use of mechanical obturators. The consideration of teeth extraction, the complications which may attend the operation, and the obstinate hemorrhage which may follow it, is very full and satisfactory. The work is copiously illustrated and its interest thereby much enhanced in the reading. The letter-press is from Spotiswoode & Co., of London, and is sharp, clear and very comfortable to the eye. We think Messrs. Wood have laid an obligation upon American surgeons by putting within their easy reach this valuable monogram.

